# Restrictive diet in a patient with irritable bowel syndrome leading to Wernicke encephalopathy

**DOI:** 10.1186/s12876-021-01758-w

**Published:** 2021-04-20

**Authors:** Qiang Wang, Zeinab Charmchi, Ilena C. George

**Affiliations:** 1 Department of Neurology, Kings County Hospital Center, SUNY Downstate Medical Center and Maimonides Medical Center, SUNY Downstate Health Sciences University, Brooklyn, NY USA; 2 Department of Neurology, Massachusetts General Hospital, 55 Fruit St, Boston, MA 02114 USA

**Keywords:** Case report, Wernicke encephalopathy, Thiamine deficiency, Irritable bowel syndrome

## Abstract

**Background:**

We present a case of a woman with a past medical history of irritable bowel syndrome (IBS) and anxiety, who presents with ophthalmoplegia, ataxia and memory loss, characteristic of Wernicke encephalopathy.

**Case presentation:**

A 64-year-old woman presented with double vision, unsteady gait and memory loss. These symptoms began after 3 months on an unfortified restricted diet, which she initiated to alleviate IBS symptoms. Magnetic resonance imaging of the brain demonstrated hyperintense T2-weighted signal in the dorsomedial aspect of bilateral thalami, periaqueductal grey matter and around the third ventricle. The patient’s visual symptoms improved significantly after thiamine supplementation, although her memory deficits persisted.

**Conclusion:**

Although WE is often associated with chronic alcohol abuse, this case demonstrates the importance of recognizing WE in any patient with a restricted diet and subsequent timely initiation of thiamine.

## Background

Wernicke encephalopathy (WE) is a neurological emergency resulting from thiamine deficiency, which impairs thiamine-dependent glucose and nucleic acid metabolism and in turn causes apoptosis due to *N*‐methyl‐d‐aspartate (NMDA) toxicity, especially in the thalamus and the mammillary bodies [1]. Although WE is most commonly seen in patients with chronic alcohol abuse, other causes include bariatric surgery, hyperemesis gravidarum and malnutrition [2]. WE is easily misdiagnosed due to clinical variance; the classic clinical triad of ocular abnormalities, ataxia, and confusion is only seen in approximately 24–38% of patients [3, 4]. Brain MRI typically reveals hyperintense signal in T2 and fluid-attenuated inversion recovery (FLAIR) and diffusion abnormality in the periaqueductal area, within the dorsomedial thalami, dorsal medulla, and mammillary bodies [5].

WE can be lethal or cause irreversible neurologic sequelae if left untreated, however, outcomes data on WE are limited. A multicenter observational study of 468 WE patients from 2000 to 2012, out of which only 34 cases were non-alcohol related, demonstrated a mortality rate during hospitalization of 5.3% [4] with a majority of these deaths due to infection or liver disease. Another study reported a 45% mortality rate after a median follow-up period of 5 years, largely due to bacterial infections and cancer [3]. Data on appropriate dosing of thiamine is similarly limited. A small case series of WE demonstrated 73% of patients had symptom improvement or resolution after rapid initiation of high dose thiamine (> 500 mg) for a median of 3 days [6].

## Case presentation

**Fig. 1:**
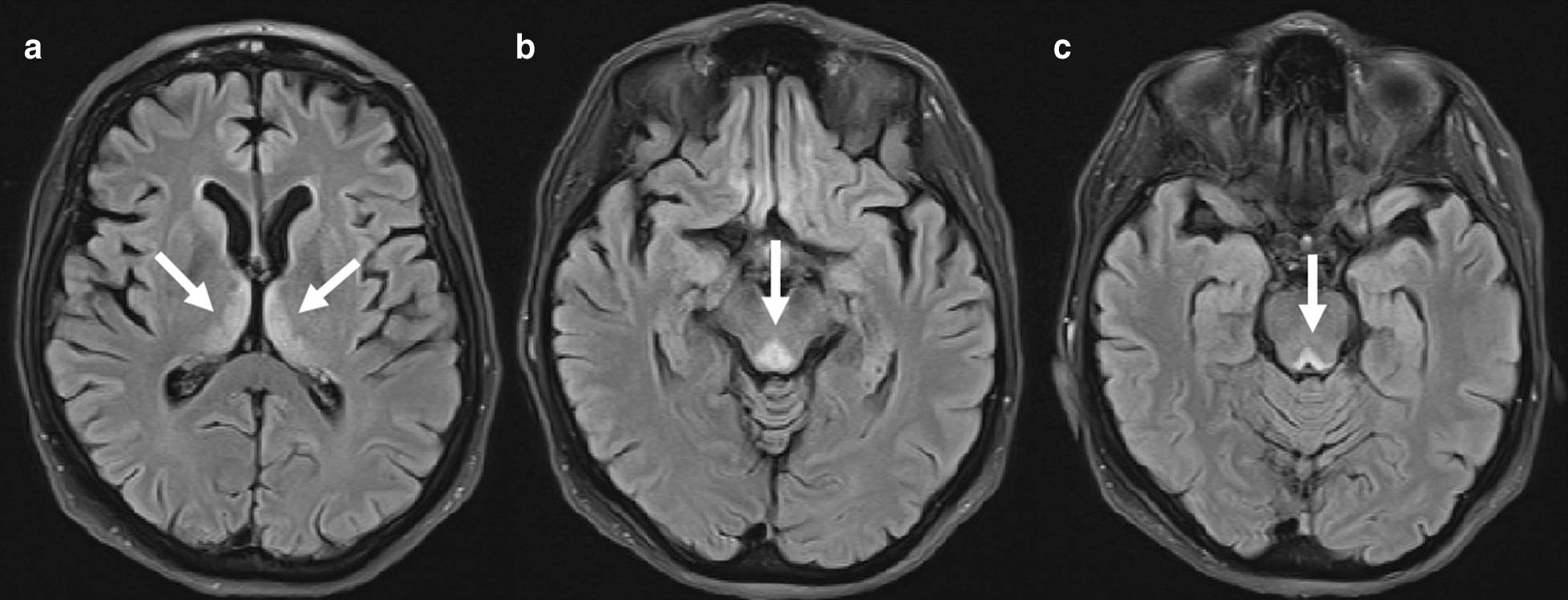
Fluid-attenuated inversion recovery imaging demonstrating characteristic changes of Wernicke encephalopathy including hyperintense signal changes in the bilateral dorsomedial thalami (**a**), inferior tectal plate (**b**) and around the periaqueductal gray matter within the dorsal midbrain (**c**)

A 64-year-old female with past medical history of hypothyroidism, irritable bowel syndrome (IBS) and panic attacks presented with double vision, unsteady gait and memory loss. She had been experiencing abdominal bloating for the past 3 months, associated with nausea and vomiting, and modified her diet to consist solely of small amounts of unfortified farina. She had similar gastrointestinal symptoms 10 years ago, for which an evaluation including endoscopy and colonoscopy was unrevealing. At that time, she was started on paroxetine for a presumptive diagnosis of IBS and her symptoms resolved after 6 months. She reported no other risk factors for thiamine deficiency, such as alcohol use, or excessive tea and/or coffee consumption. Three months prior to admission, when her symptoms recurred, her psychiatrist empirically reinitiated her on paroxetine, but discontinued this medication when her symptoms did not improve. Three weeks prior to presenting to our institution, she was noted to have double vision and inability to move her eyes laterally. A week later, she experienced a decline in her memory, and was unable to remember recent events and recognize family members. Concurrently, she developed an unsteady gait with frequent falls, which prompted an admission to a community hospital. Computed tomography of the head and abdomen were performed which were unremarkable. She was subsequently transferred to our institution for further workup including a brain MRI. The neurological exam demonstrated impaired short-term and long-term memory, near absence of horizontal eye movements with maintenance of vertical gaze, bilateral dysmetria, diminished sensation to all modalities, as well as an inability to ambulate. MRI of the brain with and without gadolinium (Fig. 1) demonstrated symmetrical hyperintense signal in fluid-attenuated inversion recovery (FLAIR) sequences around the periaqueductal grey matter, third ventricle and bilateral dorsomedial thalami. Serum thiamine level at admission was 26.8 nmol/L (normal range 66.5–200 nmol/L). Folate, vitamins B2 and B12, copper and zinc levels as well as thyroid and liver function tests were within normal range. With a clinical diagnosis of WE, she was started on high dose thiamine supplementation of 500 mg three times daily. After the first dose of thiamine, the patient’s horizontal eye movements greatly improved and returned to near-normal within 3 days of supplementation. Her memory impairment did not significantly change with thiamine administration.

## Discussion and conclusions

Wernicke encephalopathy (WE) is a relatively uncommon and potentially lethal neurological disorder with estimated prevalence between 0.8 and 2.8% [7, 8] among the general population based on autopsy studies. The actual prevalence is thought to be higher considering significant clinical variance which easily leads to misdiagnosis, however, more recent epidemiology data is limited. Proposed diagnostic criteria require two out of four signs: (a) dietary deficiencies, (b) oculomotor abnormalities, (c) cerebellar dysfunction, and (d) either an altered mental state or mild memory impairment [9]. Brain MRI can also aid in diagnosis [5]. In practice, empirical treatment of high dose thiamine has been standard management for patients with risk of nutrition deficiency who present with altered mental status, considering the low risk associated with supplementation and the extremely high risk of disease burden if misdiagnosed. The differential diagnosis is broad in such cases, including cerebrovascular event, seizures, other causes of toxic-metabolic encephalopathy, and meningoencephalitis. In cases where the diagnosis is uncertain, the workup should continue while patients receive high dose thiamine.

WE is caused by limited thiamine intake or absorption, due to conditions such as alcoholism, bariatric surgery and hyperemesis gravidarum [2]. Thiamine deficiency may alter gut microbial composition, potentially contributing to small intestine bacterial overgrowth (SIBO), a condition whose symptoms include nausea, decreased appetite, bloating, flatulence and malnutrition [10, 11]. Furthermore, IBS and SIBO are frequently co-morbid. The prevalence of SIBO increases in patients with IBS, particularly in the elderly female population [12–15]. Given her symptoms, we speculate that our patient may have had IBS/SIBO with subsequent thiamine deficiency [16], however, confirmatory testing of SIBO with a breath test or jejunal aspirate was not completed. From the available testing, the patient appeared to have an isolated thiamine deficiency as a result of her restrictive diet of unfortified farina. Unfortified farina contains common minerals including iron and vitamin D, whereas fortified farina contains additional vitamins including thiamine and niacin.

In this case, we highlight the importance of early detection and treatment of thiamine deficiency and the need for a high index of suspicion in patients with restricted diets to prevent permanent neurological sequelae. Moreover, it is paramount for patients on a restricted diet to incorporate a supplemental multivitamin including thiamine on a daily basis to prevent nutritional deficiency.

## Data Availability

The data presented in this case report is available from the corresponding author on reasonable request.

## Abbreviations

IBS: Irritable bowel syndrome
FLAIR: Fluid-attenuated inversion recovery
GI: Gastrointestinal
MRI: Magnetic resonance imaging
SIBO: Small intestine bacterial overgrowth
WE: Wernicke’s encephalopathy
